# Perceptual and Ventilatory Responses to Hypercapnia in Athletes and Sedentary Individuals

**DOI:** 10.3389/fphys.2022.820307

**Published:** 2022-03-15

**Authors:** Olivia K. Harrison, Bruce R. Russell, Kyle T. S. Pattinson

**Affiliations:** ^1^Department of Psychology, University of Otago, Dunedin, New Zealand; ^2^Nuffield Department of Clinical Neurosciences, University of Oxford, Oxford, United Kingdom; ^3^Wellcome Centre for Integrative NeuroImaging, University of Oxford, Oxford, United Kingdom; ^4^School of Pharmacy, University of Otago, Dunedin, New Zealand

**Keywords:** ventilation, perception, breathlessness, anxiety, athletes, hypercapnic chemosensitivity

## Abstract

**Purpose:**

Hypercapnic chemosensitivity traditionally captures the ventilatory response to elevated pressures of carbon dioxide in the blood. However, hypercapnia also contributes to subjective breathing perceptions, and previously we demonstrated a closer matching of perception to changes in ventilation in athletes compared to controls. Here we investigated any potential underlying hypercapnic chemosensitivity differences between groups, and explored whether these measures relate to ventilatory and perceptual responses during exercise as well as trait levels of affect.

**Methods:**

A hypercapnic challenge, incremental maximal exercise test and affective questionnaires were completed by 20 endurance athletes and 20 age-/sex-matched sedentary controls. The hypercapnic challenge involved elevating end-tidal PCO_2_ by 0.8% (6.1 mmHg) and 1.5% (11.2 mmHg) for 3 min each (randomised), with constant end-tidal oxygen. Ventilatory and perceptual responses to hypercapnia were compared between groups, and within each group the relationships between hypercapnic chemosensitivity (slope analyses) and exercising ventilation and perceptions were calculated using Spearman’s non-parametric correlations.

**Results:**

While absolute ventilation differences during hypercapnia and exercise were observed, no group differences were found across hypercapnic chemosensitivity (slope) measures. Correlation analyses revealed the anxiety hypercapnic response was related to maximal exercise anxiety, but only in sedentary individuals.

**Conclusion:**

Ventilatory and perceptual hypercapnic chemosensitivity do not differ between athletes and sedentary individuals. However, ventilatory and anxiety hypercapnic chemosensitivities were related to ventilatory and anxiety responses during exercise in untrained individuals only. Athletes may employ additional strategies during exercise to reduce the influence of chemosensitivity on ventilatory and perceptual responses.

## Introduction

Hypercapnia occurs when there is elevated pressure of carbon dioxide in the blood (PCO_2_). Increases in metabolic rate due to physical activity or exercise will increase the cellular production of CO_2_, and resulting PCO_2_. To mitigate the acidic nature of this elevated PCO_2_, chemoreceptors in the brainstem and periphery (carotid and aortic bodies) tightly control cerebral blood flow and drive ventilation (hyperpnea) to exhale excess CO_2_ (Feldman et al., 2003; Ainslie and Duffin, 2009; Ogoh et al., 2009). However, there is a broad range of variability in the magnitude of an individual’s ventilatory response to hypercapnia (Griez et al., 1990; Houtveen et al., 2003; Li et al., 2006; Peebles et al., 2007; Ogoh et al., 2008; Faull et al., 2016, 2019; Sackett et al., 2018). Furthermore, while hypercapnic chemosensitivity is a large contributing factor to ventilatory control during exercise, additional drivers such as central command output, muscle afferent feedback (Turner et al., 1997; Dempsey and Smith, 2014) and even associative conditioning (Turner and Sumners, 2002; Turner and Stewart, 2004) can influence ventilatory patterns.

Alongside hypercapnia-induced changes in ventilation, elevated PCO_2_ can also drive perceptions of both breathlessness (Banzett et al., 1990, 2008; Lane and Adams, 1993; Society, 1999; Lansing et al., 2009) and anxiety (Griez et al., 1990; Smoller et al., 1996; Houtveen et al., 2003; Johnson et al., 2012; Goossens et al., 2014). Importantly, increased ventilation due to hypercapnia does not directly translate to increased perceptions of breathlessness and anxiety (Banzett et al., 1990; Li et al., 2006), and previously we demonstrated a stronger relationship between hypercapnia-induced changes in ventilation and breathing perceptions (breathlessness and anxiety) in athletes compared to sedentary controls at rest (Faull et al., 2016). This raises the question as to whether there is an inherent difference in hypercapnic chemosensitivity in the ventilatory and/or perceptual domains in athletes, and how these responses at rest may translate to differences in ventilation and perceptions during exercise. Understanding these relationships will help shed light on the contribution of baseline ventilatory and perceptual hypercapnic chemosensitivities to our responses during incremental exercise.

Finally, exercise has been associated with reduced levels of affective traits such as anxiety and depression (Herring et al., 2011, 2014), while enhanced hypercapnic perceptions have been reported in individuals with greater trait anxiety (Li et al., 2006), panic disorder (Griez et al., 1990) and those with increased somatic symptoms (Houtveen et al., 2003). Therefore, one mechanism underlying the reduction in negative affect with regular exercise may be *via* decreasing subjective perceptual sensitivity to hypercapnia, possibly due to repeated interoceptive exposure to elevated PCO_2_ during exercise (Meuret et al., 2018). Exploring the relationship between perceptual sensitivity to hypercapnia, exercise exposure and measures such as anxiety and depression may help shed light on this effect.

Here, we utilised the athlete and sedentary groups from Faull et al. (2016) to investigate any differences in hypercapnic chemosensitivity for both ventilation and subjective perceptions. Additionally, we explored how chemosensitivity measures relate to ventilatory and perceptual responses during exercise, as well as trait measures of anxiety, depression and anxiety sensitivity (anxiety toward bodily symptoms).

## Methods

The data used for these analyses were collected as part of a wider study that considered both the physiological and functional brain response to breathlessness (Faull et al., 2016, 2018). Data pertaining to the hypercapnic challenge, incremental exercise test and questionnaires were utilised here.

### Participants

Two groups of individuals were recruited into this study, with 20 endurance athletes and 20 age- and sex-matched sedentary controls (10 males and 10 females in each group; mean age ± SD, 26 ± 7 years). Endurance athletes completed five or more training sessions per week in either running, cycling or rowing, while sedentary individuals were not involved in any organised sport and minimal commuting activity. One athlete did not complete the maximal exercise test due to injury. The Oxfordshire Clinical Research Ethics Committee approved the study and volunteers gave written, informed consent prior to participation. Participant anthropometrics are reported in Table 1.

**TABLE 1:** Participant anthropometrics.

	Athletes	Sedentary	*p*-value
Females/Males	10/10	10/10	NA
Training volume (hours/week)	11.5 ± 0.2	0.0 ± 0.0	NA
Age (years)	25.8 ± 1.7	25.7 ± 1.7	0.95
Height (m)	1.8 ± 0.2	1.7 ± 0.0	0.01*
Weight (kg)	75.2 ± 2.3	68.7 ± 3.0	0.09
BMI (kg/m^2^)	23.1 ± 0.6	23.3 ± 0.8	0.87
FVC (L)	5.7 ± 0.2	4.2 ± 0.3	<0.01*
FVC predicted (L)	5.2 ± 0.2	4.7 ± 0.2	0.10
FVC (% predicted)	108.3 ± 2.0	90.9 ± 4.3	<0.01*
FEV1 (L)	4.4 ± 0.2	3.4 ± 0.2	<0.01*
FEV1 predicted (L)	4.4 ± 0.2	4.0 ± 0.2	0.10
FEV1 (% predicted)	100.5 ± 2.1	90.9 ± 4.3	<0.01*
FEV1/FVC (%)	78.2 ± 1.6	81.3 ± 1.0	0.10
MVV (L/min)	150.9 ± 9.6	113.0 ± 8.8	0.01*
MVV predicted (L/min)	182.0 ± 6.3	144.8 ± 8.0	<0.01*
MVV (% predicted)	82.3 ± 3.5	77.7 ± 3.6	0.36
Trait anxiety	29.6 ± 1.3	30.8 ± 1.5	0.54
Anxiety sensitivity index	13.5 ± 1.4	16.1 ± 1.7	0.24
Depression	6.4 ± 0.9	7.6 ± 1.1	0.40

Data adapted from Faull et al. (2016). Mean ± SE reported for each group. BMI, body mass index; FVC, forced vital capacity; FEV1, forced expiratory volume in 1s; FEV1/FVC, forced expiratory volume in 1s as a fraction of forced vital capacity; MVV, maximal voluntary ventilation. Predicted values for FVC and FEV1 were calculated using Global Lung Index reference values (Global Lung Function Initiative, 2021; Hall et al., 2021), and predicted values for MVV were calculated with reference to FEV1 (Neder et al., 1999). *Significantly different (p < 0.05) between groups.

### Questionnaires

Participants completed questionnaires to measure anxiety (Spielberger State-Trait Anxiety Inventory; STAI) (Spielberger, 2010; Vitasari et al., 2011; Thomas and Cassady, 2021), depression (Centre for Epidemiologic Studies Depression Scale; CES-D) (Radloff, 1977; Weissman et al., 1977) and anxiety sensitivity, which measures anxiety toward anxiety symptoms (Anxiety Sensitivity Index; ASI) (Reiss et al., 1986; Maller and Reiss, 1987). Questionnaires were completed on paper and were scored according to their respective manuals.

### Spirometry

Participants additionally completed baseline spirometry measures as part of the wider study protocol. Participants breathed through a mouth-piece (Hans Rudolf, Kansas City, MO, United States) and turbine connected to gas and flow analyser (Cortex Metalyser 3B, Cranlea Human Performance Ltd., Birmingham, United Kingdom) while wearing a nose clip. Metasoft studio software (Cortex, Versions 3.9.9 and 4.9.0, Cranlea Human Performance Ltd., Birmingham, United Kingdom) was used to calculate all spirometry measurements. Forced vital capacity (FVC) and Fraction of Expired Volume in 1 s (FEV1) were measured using a full inspiration and expiration, repeated three times in accordance to established guidelines (Levy et al., 2009). Spirometry protocols matched those defined in the American Thoracic Society and European Respiratory Society 2019 update for usable tests (Graham et al., 2019), although FVC measures were not followed by a full inspiration. The best of two repeats of maximal voluntary ventilation (MVV) were recorded, where participants were asked to maximally ventilate through the mouthpiece for 10 s.

### Hypercapnic Challenge

Participants were positioned supine and asked to breathe through a custom-built gas mixing circuit *via* a mouthpiece (Scubapro United Kingdom Ltd., Mitcham, United Kingdom) connected to a bacterial and viral filter (GVS, Lancashire, United Kingdom) whilst wearing a nose clip. Participants were given prism glasses such that they could see and respond to questions presented on a computer screen *via* a button box throughout the task. The breathing circuit allowed for measures of end-tidal pressure of oxygen (P_ET_O_2_) and carbon dioxide (P_ET_CO_2_) *via* polyethylene extension tubing (Vygon SA, Ecouen, France) connected to a gas analyser (ADInstruments Ltd., Oxford, United Kingdom). A spirometer (ADInstruments Ltd., Oxford, United Kingdom) simultaneously measured ventilatory flow and volume, and all devices were connected to a data acquisition device (Powerlab; ADInstruments Ltd., Oxford, United Kingdom) with measures recorded using physiological monitoring software (Labchart 7; ADInstruments Ltd., Oxford, United Kingdom).

Following 8 min of rest where participants breathed humidified medical air, two three-minute hypercapnic periods of elevated P_ET_CO_2_, either 0.8% (6.1 mm Hg) or 1.5% (11.2 mm Hg) above baseline were administered (randomised order), separated and followed by 4 min of rest breathing medical air. P_ET_CO_2_ values were chosen to induce two distinguishable levels of hypercapnia within a tolerable range for all participants, and hypercapnia was achieved by titrating a CO_2_ mixture (25% CO_2_; 21% O_2_; balance N_2_; supplied by BOC Gas, Oxford, United Kingdom) into a custom-built mixing chamber and breathing circuit (see Faull et al., 2016 for details). P_ET_O_2_ was maintained at resting levels throughout the task by simultaneously titrating a hypoxic gas (7% O_2_; balance nitrogen; supplied by BOC Gas, Oxford, United Kingdom) into the inspiratory mixture. Every 4 min participants were asked to rate their breathlessness by answering the question “How breathless are you” *via* the button box between “Not at all breathless” (0%) and “Most intense breathlessness imaginable” (100%) using a visual analogue scale (VAS). Participants additionally answered the question “How anxious are you about your breathing?” using a VAS between “Not at all anxious” (0%) and “Extremely anxious” (100%) following the breathlessness rating.

### Incremental Maximal Exercise Test

Participants completed an incremental exercise test to exhaustion on a stationary bicycle ergometer (Ergoline 500, Lindenstrasse, Germany). Participants were fitted with a facemask (Hans Rudolph Inc., Kansas, United States) and turbine connected to a gas and flow analyser (Cortex Metalyser 3B, Cranlea Human Performance Ltd., Birmingham, United Kingdom) for breath-by-breath measures of expired gases and ventilatory flow. Heart rate was measured by a Polar heart rate monitor (Polar, Kempele, Finland) connected *via* Bluetooth. Exercise was initiated between 50 and 150 W according to predicted maximal effort, and cadence was self-selected cadence with an aim of 90 rpm. Three-minute stages at 50 W increments were completed until volitional exhaustion. Breathlessness and breathing-related anxiety were additionally rated on a 0–100% VAS scale verbally in the last 30 s of each stage and at exhaustion. Physiological measures were averaged across the final 30 s at each stage, and the anaerobic threshold for each participant was determined by visual inspection using the V-slope method (Wasserman et al., 1973; Beaver et al., 1986).

### Statistical Analyses

To measure both the ventilatory and perceptual responses to hypercapnia, chemosensitivity metrics for ventilation, tidal volume, breathing rate, breathlessness and anxiety of breathing were calculated. A separate linear model was fit for each of these measures against P_ET_CO_2_ during the hypercapnic challenge, and the slope coefficient (representing the rate of change in each of the metrics according to the mm Hg increase in P_ET_CO_2_) was used as the subsequent hypercapnic chemosensitivity metric. Following tests for data normality (Anderson-Darling test, with an alpha value of *p* < 0.05 rejecting the null hypothesis of normally distributed data), the slope parameter for each of the measures was compared between athlete and sedentary groups using two-tailed independent *t*-tests. If the data were not normally distributed, significant group differences were tested using non-parametric Wilcoxon rank sum tests. To account for the multiple group comparison tests, we utilised False Discovery Rate (FDR)-corrected significance values at *p* < 0.05, with values surviving *p* < 0.05 uncorrected reported as exploratory results. Tidal volume and breathing rate during both the hypercapnic challenge and exercise were also compared between the groups, in addition to the comparisons previously reported by Faull et al. (2016).

To compare each of the hypercapnic chemosensitivity measures with both exercising variables (ventilation, breathlessness and anxiety scores at anaerobic threshold and maximal exercise) and questionnaire scores relating to affect (anxiety, depression, anxiety sensitivity), we constructed a full correlation matrix of these variables for each of the athlete and sedentary groups. To reduce the impact of outliers with only 20 participants in each group, we employed non-parametric Spearman correlations, with significance taken as correlation coefficients having a *p* < 0.05 with FDR correction for multiple comparisons across the correlation matrix. Values surviving *p* < 0.05 uncorrected are reported as exploratory results.

## Results

### Hypercapnic Chemosensitivity

**FIGURE 1:**
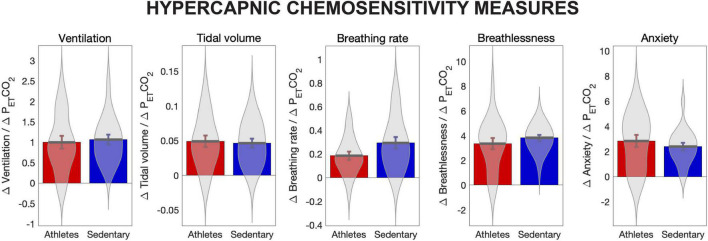
Hypercapnic chemosensitivity measures for athletes and sedentary controls. Hypercapnic chemosensitivity for ventilation, tidal volume, breathing rate, perception of breathlessness and perception of breathing-related anxiety are the rate of change in each value for each mm Hg increase in P_ET_CO_2_, calculated using the slope parameter from a fitted linear model. Ventilation, tidal volume and breathing rate were normalised to height. No significant differences were observed between groups for any chemosensitivity measures.

Hypercapnic chemosensitivity values (slope parameter for the change in each metric in response to increases in P_ET_CO_2_) for ventilation, tidal volume, breathing rate, breathlessness and anxiety of breathing are provided in Figure 1. Ventilation, tidal volume and breathing rate were normalised to height. No significant differences were observed between athletes and sedentary groups.

A summary of the ventilatory and perceptual responses to the hypercapnic challenge for athletes and sedentary groups can be seen in Table 2. As reported previously (Faull et al., 2016), athletes and sedentary individuals were found to differ at rest for ventilation. Additionally, here we have found differences in tidal volume at rest (mean ± SE: athletes 1.37 ± 0.14 L vs. sedentary 0.83 ± 0.07 L; *z* = 3.19; *p* < 0.01; Wilcoxon rank sum) and both mild hypercapnia (mean ± SE: athletes 1.99 ± 0.18 L vs. sedentary 1.26 ± 0.10 L; *z* = 3.02; *p* < 0.01; Wilcoxon rank sum) and moderate hypercapnia (mean ± SE: athletes 2.39 ± 0.18 L vs. sedentary 1.74 ± 0.17 L; *t* = 2.62; *p* = 0.01; *t*-test), as well as differences in breathing rate at rest (mean ± SE: athletes 10.47 ± 0.70 bpm vs. sedentary 13.79 ± 0.90 bpm; *t* = −2.91; *p* = 0.01; *t*-test) and both mild hypercapnia (mean ± SE: athletes 12.30 ± 0.95 bpm vs. sedentary 17.71 ± 1.29 bpm; *z* = −3.15; *p* < 0.01; Wilcoxon rank sum) and moderate hypercapnia (mean ± SE: athletes 14.42 ± 0.96 bpm vs. sedentary 19.20 ± 1.30 bpm; *t* = −2.94; *p* = 0.01; *t*-test).

**TABLE 2:** Ventilatory and perceptual responses to a hypercapnic challenge for athletes and sedentary controls.

Rest	Athletes	Sedentary	t/z statistic	*p*-value	Test
P_ET_CO_2_ (mm Hg)	38.9 ± 1.1	40.1 ± 0.8	−0.45	0.66	Wxn
P_ET_O_2_ (mm Hg)	116.3 ± 1.7	115.7 ± 1.5	0.27	0.79	Ttest
Ventilation (L/min)	13.0 ± 0.8	10.5 ± 0.6	2.34	0.02*	Wxn
Ventilation/height (L/min/m)	7.2 ± 0.4	6.1 ± 0.3	1.99	0.05	Ttest
Tidal volume (L)	1.4 ± 0.1	0.8 ± 0.1	3.19	<0.01*	Wxn
Tidal volume/height (L/m)	0.8 ± 0.1	0.5 ± 0.0	3.02	<0.01*	Wxn
Breathing rate (bpm)	10.5 ± 0.7	13.8 ± 0.9	−2.91	0.01*	Ttest
Breathing rate/height (bpm/m)	5.8 ± 0.4	8.2 ± 0.6	−3.16	<0.01*	Ttest
Breathlessness rating (%)	3.1 ± 1.0	4.6 ± 0.9	−1.66	0.10	Wxn
Breathing anxiety rating (%)	3.1 ± 0.9	5.1 ± 1.1	−1.56	0.12	Wxn

**Hypercapnia: Mild**	**Athletes**	**Sedentary**	**t/z statistic**	***p*-value**	**Test**

P_ET_CO_2_ (mm Hg)	46.0 ± 1.0	46.5 ± 0.7	0.15	0.88	Wxn
P_ET_O_2_ (mm Hg)	114.7 ± 1.2	115.8 ± 1.0	−0.70	0.49	Ttest
Ventilation (L/min)	23.3 ± 2.3	20.6 ± 1.1	0.45	0.66	Ttest
Ventilation/height (L/min/m)	12.9 ± 1.3	12.0 ± 0.6	−0.07	0.95	Wxn
Tidal volume (L)	2.0 ± 0.2	1.3 ± 0.1	3.02	<0.01*	Wxn
Tidal volume/height (L/m)	1.1 ± 0.1	0.7 ± 0.1	2.77	0.01*	Wxn
Breathing rate (bpm)	12.3 ± 0.9	17.7 ± 1.3	−3.15	<0.01*	Wxn
Breathing rate/height (bpm/m)	6.9 ± 0.6	10.5 ± 0.9	−3.12	<0.01*	Wxn
Breathlessness rating (%)	26.0 ± 4.5	21.9 ± 3.5	0.72	0.48	Ttest
Breathing anxiety rating (%)	18.9 ± 4.0	17.2 ± 3.1	−0.23	0.82	Wxn

**Hypercapnia: moderate**	**Athletes**	**Sedentary**	**t/z statistic**	***p*-value**	**Test**

P_ET_CO_2_ (mm Hg)	50.6 ± 1.1	51.2 ± 0.7	−0.49	0.63	Ttest
P_ET_O_2_ (mm Hg)	115.9 ± 1.0	117.6 ± 1.3	−1.00	0.32	Ttest
Ventilation (L/min)	34.0 ± 3.3	31.1 ± 2.6	0.70	0.49	Ttest
Ventilation/height (L/min/m)	18.8 ± 7.9	18.1 ± 1.4	0.33	0.74	Ttest
Tidal volume (L)	2.4 ± 0.2	1.7 ± 0.2	2.62	0.01*	Ttest
Tidal volume/height (L/m)	1.3 ± 0.1	1.0 ± 0.1	2.47	0.02*	Ttest
Breathing rate (bpm)	14.4 ± 1.0	19.2 ± 1.3	−2.94	0.01*	Ttest
Breathing rate/height (bpm/m)	8.0 ± 0.6	11.4 ± 0.9	−2.77	0.01*	Wxn
Breathlessness rating (%)	41.9 ± 5.4	47.8 ± 2.8	−0.69	0.49	Wxn
Breathing anxiety rating (%)	36.5 ± 5.8	32.3 ± 3.6	0.62	0.54	Ttest

**Exercise: Anaerobic threshold**	**Athletes**	**Sedentary**	**t/z statistic**	***p*-value**	**Test**

Work rate (W)	219.7 ± 10.5	101.3 ± 5.6	5.26	<0.01*	Wxn
VO_2_ (mL/min/kg)	36.5 ± 2.5	20.3 ± 1.0	5.91	<0.01*	Ttest
P_ET_CO_2_ (mm Hg)	41.6 ± 0.8	40.7 ± 0.8	0.77	0.45	Ttest
P_ET_O_2_ (mm Hg)	108.4 ± 1.0	109.2 ± 0.9	−0.59	0.56	Ttest
Ventilation (L/min)	79.7 ± 3.8	38.6 ± 2.2	4.96	<0.01*	Wxn
Ventilation/height (L/min/m)	42.1 ± 1.9	22.4 ± 1.2	4.99	<0.01*	Wxn
Tidal volume (L)	2.6 ± 0.1	1.5 ± 0.1	5.76	<0.01*	Ttest
Tidal volume/height (L/m)	1.4 ± 0.1	0.8 ± 0.1	6.11	<0.01*	Ttest
Breathing rate (bpm)	30.5 ± 1.1	28.6 ± 1.8	0.85	0.40	Ttest
Breathing rate/height (bpm/m)	16.8 ± 0.6	17.0 ± 1.2	−0.09	0.93	Ttest
Heart rate (bpm)	152.7 ± 3.2	135.2 ± 3.8	3.47	<0.01*	Ttest
Breathlessness rating (%)	22.9 ± 3.8	16.1 ± 3.0	1.30	0.19	Wxn
Breathing anxiety rating (%)	5.9 ± 1.8	5.1 ± 2.1	0.69	0.49	Wxn

**Exercise: Maximum**	**Athletes**	**Sedentary**	**t/z statistic**	***p*-value**	**Test**

Work rate (W)	325.0 ± 13.3	173.8 ± 10.2	8.94	<0.01*	Ttest
VO_2_ (mL/min/kg)	50.8 ± 1.6	31.6 ± 1.6	8.22	<0.01*	Ttest
P_ET_CO_2_ (mm Hg)	33.2 ± 1.0	35.3 ± 0.8	−1.69	0.10	Ttest
P_ET_O_2_ (mm Hg)	120.1 ± 1.2	117.9 ± 0.8	1.52	0.14	Ttest
Ventilation (L/min)	146.4 ± 8.2	77.8 ± 6.0	6.63	<0.01*	Ttest
Ventilation/height (L/min/m)	80.5 ± 4.2	44.7 ± 3.0	6.86	<0.01*	Ttest
Tidal volume (L)	2.8 ± 0.1	1.9 ± 0.2	3.82	<0.01*	Wxn
Tidal volume/height (L/m)	1.6 ± 0.1	1.1 ± 0.1	3.78	<0.01*	Wxn
Breathing rate (bpm)	52.5 ± 2.6	41.4 ± 1.4	2.74	0.01*	Wxn
Breathing rate/height (bpm/m)	28.9 ± 1.4	24.3 ± 0.9	2.77	0.01*	Ttest
Heart rate (bpm)	180.2 ± 1.7	172.9 ± 2.9	2.04	0.05*	Ttest
Breathlessness rating (%)	80.7 ± 5.1	72.5 ± 3.8	1.91	0.06	Wxn
Breathing anxiety rating (%)	45.3 ± 8.1	22.3 ± 4.5	1.89	0.06	Wxn

Values were taken at rest and two levels of hypercapnia (0.8% and 1.5% increases in P_ET_CO_2_) while P_ET_O_2_ was held constant (iso-oxia). Mean ± SE reported for each group. *Significant differences between groups, compared using either an unpaired T-test (Ttest) if data were normally distributed or a Wilcoxon rank sum test (Wxn) if data were not normally distributed. Data adapted from (Faull et al., 2016).

### Ventilation and Perception During Exercise

A summary of the ventilatory and perceptual responses at both anaerobic threshold and maximal exercise for athletes and sedentary groups can be seen in Table 2. As reported previously (Faull et al., 2016), athletes and sedentary individuals were found to differ at both anaerobic threshold and maximal exercise for ventilation, and anxiety of breathing was greater in athletes at maximal exercise. Additionally, here we found differences in tidal volume at anaerobic threshold (mean ± SE: athletes 2.56 ± 0.14 L vs. sedentary 1.47 ± 0.13 L; *t* = 5.76; *p* < 0.01; *t*-test) and maximal exercise (mean ± SE: athletes 2.82 ± 0.10 L vs. sedentary 1.92 ± 0.15 L; *z* = 3.82; *p* < 0.01; Wilcoxon rank sum), and also for breathing rate at maximal exercise (mean ± SE: athletes 52.46 ± 2.57 bpm vs. sedentary 41.38 ± 1.38 bpm; *z* = 2.74; *p* = 0.01; Wilcoxon rank sum) but not anaerobic threshold (mean ± SE: athletes 30.45 ± 1.13 bpm vs. sedentary 28.63 ± 1.79 bpm; *t* = 0.85; *p* = 0.40; *t*-test).

### Correlations Between Hypercapnic Chemosensitivity, Exercise and Affect

**FIGURE 2:**
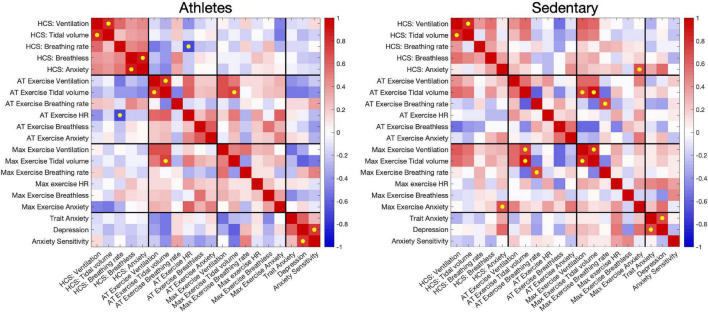
Correlations between ventilatory and perceptual hypercapnic chemosensitivity measures, exercising ventilation, heart rate and perceptions, and affective measures of anxiety, depression and anxiety sensitivity. Correlations were performed separately for athletes and sedentary groups. Yellow dots denote correlations that have *p* < 0.05 and are FDR-corrected for multiple comparisons. HCS = Hypercapnic chemosensitivity; AT = Anaerobic threshold, determined using the V-slope method (Wasserman et al., 1973; Beaver et al., 1986).

A full correlation matrix was calculated between hypercapnic chemosensitivity measures, exercise and affect values for each of the athlete and sedentary groups (Figure 2). As seen in Figure 2, athletes demonstrated stronger and more consistent correlations between hypercapnic chemosensitivity metrics than sedentary individuals. Both athletes and sedentary groups showed strong correlations between anaerobic threshold and maximal exercising ventilations and tidal volumes, with breathlessness and anxiety ratings correlated at anaerobic threshold for both groups.

A significant relationship was also seen between hypercapnic chemosensitivity of breathing anxiety ratings and maximal exercise breathing anxiety in sedentary participants. Additionally, breathlessness ratings at anaerobic threshold were inversely correlated with maximal exercise ventilation in the sedentary group. For the athletes, a significant inverse relationship was found between hypercapnic reactivity for tidal volume and heart rate at anaerobic threshold.

For both groups, anxiety and depression scores were closely correlated. Additionally, a relationship was observed between depression and ASI affective scores in the athlete group, which was not apparent in the sedentary group. The affective scores did not correlate strongly with any other exercise or hypercapnic measures, although several weaker relationships were observed with these measures (see Figure 2).

## Discussion

### Main Findings

Hypercapnic chemosensitivity has typically been measured as the change in ventilation in response to a hypercapnic challenge (Peebles et al., 2007; Ogoh et al., 2008; Faull et al., 2016; Sackett et al., 2018). Here, we extended this to include the perceptual responsivity to hypercapnia using ratings of breathlessness and anxiety toward breathing. As only weak relationships were observed between ventilatory and perceptual responsivity parameters, these measures appeared to be largely independent. There were no differences in any of the measured hypercapnic chemosensitivity responses (ventilation, tidal volume, breathing rate, breathlessness and anxiety of breathing) between athletes and sedentary controls. However, different ventilatory strategies were found during the hypercapnic challenge, with athletes utilising larger tidal volumes and lower breathing rates during hypercapnia (adjusted for height differences between groups), despite no differences in overall ventilation. Athletes also recorded greater work rate and volume of oxygen consumption (VO_2_) as expected, and correspondingly greater ventilation during sub-maximal and maximal exercise.

Additionally, the relationship between hypercapnic chemosensitivity and exercising ventilation and perceptions differed between groups. Sedentary individuals demonstrated a strong relationship between hypercapnic chemosensitivity of anxiety at rest and breathing anxiety during maximal exercise, while athletes demonstrated a strong inverse relationship where greater hypercapnic chemosensitivity of breathing rate was related to lower heart rate during exercise at anaerobic threshold. Finally, no hypercapnic nor exercising ventilatory parameters or perceptions were related to affective traits of anxiety, depression and anxiety sensitivity.

### Hypercapnic Chemosensitivity and Exercise

Here we have shown that hypercapnic chemosensitivity appears to be related to exercising perceptions of anxiety in sedentary individuals. This means that those who have a greater anxiety response to hypercapnia also report greater perceptions of breathing anxiety when exercising at maximal intensity. As this relationship was not observed in athletes, it is possible that training may allow factors such as increased motor drive and conditioned responses (Turner et al., 1997; Turner and Sumners, 2002; Turner and Stewart, 2004; Dempsey and Smith, 2014) to override some of the effects of perceptual hypercapnic chemosensitivity during exercise. Conversely, in athletes, greater hypercapnic chemosensitivity of breathing rate was strongly related to lower heart rate during sub-maximal exercise. This possibly reflects a compensatory mechanism whereby smaller hypercapnia-stimulated changes in breathing rate can be accounted for by larger increases in heart rate during exercise, both of which can act to maintain arterial blood gas homeostasis (Meersman et al., 1995; Convertino, 2019).

Despite no differences in hypercapnic chemosensitivity measures between the groups, we did observe marked discrepancies in the ventilatory strategies employed during the hypercapnic challenge. During the ventilatory response to hypercapnia, tidal volume was greater and breathing rate lower in athletes, although ventilatory responses overall were similar between groups. These differences in ventilatory patterns remained after standardisation against height, and percentage of predicted values for FVC and FEV1 were also lower in sedentary individuals. While exercise training typically results in limited changes in lung capacity measures but improvements in measures of lung function (Dunham and Harms, 2012; Khosravi et al., 2013), these differences may be due to a combination of training and a self-selection bias, where individuals with better ventilatory capacity choose to participate in endurance sports (which improves lung function), resulting in greater tidal volumes and lower breathing rates during ventilation.

Finally, there were no relationships between hypercapnic chemosensitivity nor exercising parameters with trait measures of anxiety, depression or anxiety sensitivity. Notably, athletes demonstrated a strong relationship between depression and anxiety sensitivity (fear of anxiety symptoms) that was not present in sedentary individuals, while overall scores for both measures were similar between groups. This may be related to a greater awareness and anticipation of body symptoms in athletes (Faull et al., 2018), although further research is required to understand the effects of exercise training on perception of anxiety symptoms in the body.

### Limitations

This study is a supplementary analysis of previously published work and is exploratory in nature. A number of limitations must be addressed in further work in this area, beginning with testing participants in consistent postures across exercise and hypercapnic chemosensitivity measures. Here, participants underwent the hypercapnic challenge while supine (for ease of for ease of use of the custom-built breathing system designed to deliver hypercapnic stimuli), while exercise was undertaken seated on a bicycle ergometer. Postural differences are known to affect lung function measures (Allen et al., 1985), and thus these differences may have confounded the results in the current study.

Secondly, limited physiological data were available in this study. Future work may look to incorporate measures of blood lactate, blood pressure and/or oxygen saturation measures to better understand the physiological and perceptual responses to hypercapnic stimuli in athletes and sedentary controls. Additionally, female participants were not tested in the same part of their menstrual phase, likely adding variability to the physiological and perceptual responses recorded in this dataset.

Finally, the correlations reported here cannot be assumed to infer causation. The results of this study can only provide us with an overview as to the possible relationships between hypercapnic chemosensitivity and exercising physiology and perceptions. Further research is required using perturbations (such as hypercapnic and hypoxic stimuli *during* exercise), such that the influence of hypercapnic chemosensitivity directly on exercising parameters can be inferred.

## Conclusion

Hypercapnic chemosensitivity does not appear to be altered in athletes compared to sedentary individuals, either in the ventilatory or perceptual domains. Multiple relationships exist between hypercapnic chemosensitivity and exercising ventilation/perceptions in sedentary individuals but not athletes, which may be due to exercise training or self-selection biases. Sedentary individuals may use both ventilatory and perceptual responses to hypercapnia to constrain their exercising performance, while athletes may override these signals using factors such as goal-directed increases in motor output.

## Data Availability Statement

The raw data supporting the conclusions of this article will be made available by the authors, without undue reservation.

## Ethics Statement

The studies involving human participants were reviewed and approved by Oxfordshire Clinical Research Ethics Committee. The patients/participants provided their written informed consent to participate in this study.

## Author Contributions

OH and KP contributed to conception and design of the study. OH collected the data and performed the statistical analyses. OH wrote the first draft of the manuscript, with advice from KP and BR. All authors contributed to manuscript revision, read, and approved the submitted version.

## Conflict of Interest

The authors declare that the research was conducted in the absence of any commercial or financial relationships that could be construed as a potential conflict of interest.

## Publisher’s Note

All claims expressed in this article are solely those of the authors and do not necessarily represent those of their affiliated organizations, or those of the publisher, the editors and the reviewers. Any product that may be evaluated in this article, or claim that may be made by its manufacturer, is not guaranteed or endorsed by the publisher.

## References

[B1] Ainslie P. N. Duffin J. (2009). Integration of cerebrovascular CO2 reactivity and chemoreflex control of breathing: mechanisms of regulation, measurement, and interpretation. *Am. J. Physiol.-Regul. Integr. Comp. Physiol* 296 R1473–R1495. 10.1152/ajpregu.91008.2008 19211719

[B2] Allen S. M. Hunt B. Green M. (1985). Fall in vital capacity with posture. *Brit. J. Dis. Chest* 79 267–271. 10.1016/0007-0971(85)90047-6 4015957

[B3] Banzett R. B. Lansing R. W. Brown R. Topulos G. P. (1990). ‘Air hunger’from increased PCO2 persists after complete neuromuscular block in humans. *Respir. Physiol.* 81 1–17. 10.1016/0034-5687(90)90065-7 2120757

[B4] Banzett R. B. Pedersen S. H. Schwartzstein R. M. Lansing R. W. (2008). The Affective Dimension of Laboratory Dyspnea. *Am. J. Respir. Critic. Care Med.* 177 1384–1390. 10.1164/rccm.200711-1675OC 18369200 PMC2427058

[B5] Beaver W. L. Wasserman K. Whipp B. J. (1986). A New Method for Detecting Anaerobic Threshold by Gas-Exchange. *J. Appl. Physiol.* 60 2020–2027. 10.1152/jappl.1986.60.6.2020 3087938

[B6] Convertino V. A. (2019). Mechanisms of inspiration that modulate cardiovascular control: the other side of breathing. *J. Appl. Physiol.* 127 1187–1196. 10.1152/japplphysiol.00050.2019 31225967

[B7] Dempsey J. A. Smith C. A. (2014). Pathophysiology of human ventilatory control. *Euro. Respir. J.* 44 495–512. 10.1183/09031936.00048514 24925922 PMC4578297

[B8] Dunham C. Harms C. A. (2012). Effects of high-intensity interval training on pulmonary function. *Europ. J. Appl. Physiol.* 112 3061–3068. 10.1007/s00421-011-2285-5 22194005

[B9] Faull O. K. Cox P. J. Pattinson K. T. S. (2016). Psychophysical Differences in Ventilatory Awareness and Breathlessness between Athletes and Sedentary Individuals. *Front. Physiol.* 7:231. 10.3389/fphys.2016.00231 27378940 PMC4910254

[B10] Faull O. K. Cox P. J. Pattinson K. T. S. (2018). Cortical processing of breathing perceptions in the athletic brain. *Neuroimage* 179 92–101. 10.1016/j.neuroimage.2018.06.021 29890328

[B11] Faull O. K. Dearlove D. J. Clarke K. Cox P. J. (2019). Beyond RPE: the Perception of Exercise Under Normal and Ketotic Conditions. *Front. Physiol.* 10:229. 10.3389/fphys.2019.00229 30941052 PMC6433983

[B12] Feldman J. L. Mitchell G. S. Nattie E. E. (2003). BREATHING: rhythmicity. *Plasticity, Chemosensitivity*. *Ann. Rev. Neurosci.* 26 239–266. 10.1146/annurev.neuro.26.041002.131103 12598679 PMC2811316

[B13] Global Lung Function Initiative. (2021). *Global Lung Function Initiative Calculators for Spirometry, TLCO and Lung Volume.* Available online at http://gli-calculator.ersnet.org/index.html (accessed February 04, 2022).

[B14] Goossens L. Leibold N. Peeters R. Esquivel G. Knuts I. Backes W. (2014). Brainstem response to hypercapnia: a symptom provocation study into the pathophysiology of panic disorder. *J. Psychopharmacol.* 28 449–456. 10.1177/0269881114527363 24646808

[B15] Graham B. L. Steenbruggen I. Miller M. R. Barjaktarevic I. Z. Cooper B. G. Hall G. L. (2019). Standardization of Spirometry 2019 Update. An Official American Thoracic Society and European Respiratory Society Technical Statement. *Am. J. Respir. Critic. Care Med.* 200 e70–e88. 10.1164/rccm.201908-1590st 31613151 PMC6794117

[B16] Griez E. Zandbergen J. Pols H. Loof C. D. (1990). Response to 35% CO2 as a marker of panic in severe anxiety. *Am. J. Psychiatr.* 147 796–797. 10.1176/ajp.147.6.796 2111639

[B17] Hall G. L. Filipow N. Ruppel G. Okitika T. Thompson B. Kirkby J. (2021). Official ERS technical standard: global Lung Function Initiative reference values for static lung volumes in individuals of European ancestry. *Euro. Respir. J.* 57:2000289. 10.1183/13993003.00289-2020 33707167

[B18] Herring M. P. Jacob M. L. Suveg C. O’Connor P. J. (2011). Effects of short-term exercise training on signs and symptoms of generalized anxiety disorder. *Mental Health Phys. Activ.* 4 71–77. 10.1016/j.mhpa.2011.07.002

[B19] Herring M. P. Lindheimer J. B. O’Connor P. J. (2014). The Effects of Exercise Training on Anxiety. *Am. J. Lifestyle Med.* 8 388–403. 10.1177/1559827613508542

[B20] Houtveen J. H. Rietveld S. de.Geus E. J. C. (2003). Exaggerated perception of normal physiological responses to stress and hypercapnia in young women with numerous functional somatic symptoms. *J. Psychosomatic Res.* 55 481–490. 10.1016/s0022-3999(03)00011-4 14642976

[B21] Johnson P. L. Samuels B. C. Fitz S. D. Lightman S. L. Lowry C. A. Shekhar A. (2012). Activation of the Orexin 1 Receptor is a Critical Component of CO2-Mediated Anxiety and Hypertension but not Bradycardia. *Neuropsychopharmacology* 37 1911–1922. 10.1038/npp.2012.38 22453138 PMC3376323

[B22] Khosravi M. Tayebi S. M. Safari H. (2013). Single and concurrent effects of endurance and resistance training on pulmonary function. *Iran. J. Basic Med. Sci.* 16 628–634. 24250940 PMC3821882

[B23] Lane R. Adams L. (1993). Metabolic acidosis and breathlessness during exercise and hypercapnia in man. *J. Physiol.* 461 47–61. 10.1113/jphysiol.1993.sp019500 8350272 PMC1175244

[B24] Lansing R. W. Gracely R. H. Banzett R. B. (2009). The multiple dimensions of dyspnea: review and hypotheses. *Respir. Physiol. Neurobiol.* 167 53–60. 10.1016/j.resp.2008.07.012 18706531 PMC2763422

[B25] Levy M. L. Quanjer P. H. Rachel B. Cooper B. G. Holmes S. Small I. R. (2009). Diagnostic Spirometry in Primary Care. *Primary Care Respir. J.* 18 130–147. 10.4104/pcrj.2009.00054 19684995 PMC6619276

[B26] Li W. Daems E. de Woestijne K. P. V. Diest I. V. Gallego J. Peuter S. D. (2006). Air hunger and ventilation in response to hypercapnia: effects of repetition and anxiety. *Physiol. Behav.* 88 47–54. 10.1016/j.physbeh.2006.03.001 16626764

[B27] Maller R. G. Reiss S. (1987). A behavioral validation of the anxiety sensitivity index. *J. Anxie. Dis.* 1 265–272. 10.1016/0887-6185(87)90031-4

[B28] Meersman R. E. D. Reisman S. S. Daum M. Zorowitz R. Leifer M. Findley T. (1995). Influence of respiration on metabolic, hemodynamic, psychometric, and R-R interval power spectral parameters. *Am. J. Physiol. Heart and Circulatory Physiol.* 269 H1437–H1440. 10.1152/ajpheart.1995.269.4.h1437 7485578

[B29] Meuret A. E. Ritz T. Wilhelm F. H. Roth W. T. Rosenfield D. (2018). Hypoventilation Therapy Alleviates Panic by Repeated Induction of Dyspnea. *Biol. Psychiatr.: Cogn. Neurosci. Neuroimaging*, 3 1–29. 10.1016/j.bpsc.2018.01.010 29573981 PMC6019126

[B30] Neder J. A. Andreoni S. Lerario M. C. Nery L. E. (1999). Reference values for lung function tests: II. Maximal respiratory pressures and voluntary ventilation. *Brazil. J. Med. Biol. Res.* 32 719–727. 10.1590/s0100-879x1999000600007 10412550

[B31] Ogoh S. Ainslie P. N. Miyamoto T. (2009). Onset responses of ventilation and cerebral blood flow to hypercapnia in humans: rest and exercise. *J. Appl. Physiol.* 106 880–886. 10.1152/japplphysiol.91292.2008 19131474 PMC2660255

[B32] Ogoh S. Hayashi N. Inagaki M. Ainslie P. N. Miyamoto T. (2008). Interaction between the ventilatory and cerebrovascular responses to hypo- and hypercapnia at rest and during exercise. *J Physiol.* 586 4327–4338. 10.1113/jphysiol.2008.157073 18635644 PMC2652171

[B33] Peebles K. Celi L. McGrattan K. Murrell C. Thomas K. Ainslie P. N. (2007). Human cerebrovascular and ventilatory CO2 reactivity to end-tidal, arterial and internal jugular vein PCO2. *J. Physiol.* 584 347–357. 10.1113/jphysiol.2007.137075 17690148 PMC2277051

[B34] Radloff L. S. (1977). The CES-D scale a self-report depression scale for research in the general population. *Appl. Psychol. Measurement* 1 385–401. 10.1177/014662167700100306 26918431

[B35] Reiss S. Peterson R. A. Gursky D. M. McNally R. J. (1986). Anxiety sensitivity, anxiety frequency and the prediction of fearfulness. *Behav. Res. Ther.* 24 1–8. 10.1016/0005-7967(86)90143-9 3947307

[B36] Sackett J. R. Schlader Z. J. Cruz C. Hostler D. Johnson B. D. (2018). The effect of water immersion and acute hypercapnia on ventilatory sensitivity and cerebrovascular reactivity. *Physiol. Rep.* 6:e13901. 10.14814/phy2.13901 30369098 PMC6204237

[B37] Smoller J. W. Pollack M. H. Otto M. W. Rosenbaum J. F. Kradin R. L. (1996). Panic anxiety, dyspnea, and respiratory disease: theoretical and clinical considerations. *American J. Respir. Critic. Care Med.* 154 6–17. 10.1164/ajrccm.154.1.8680700 8680700

[B38] Society A. T. (1999). Dyspnea Mechanisms, Assessment, and Management: a Consensus Statement. *Am. J. Respir. Critic. Care Med.* 159 321–340. 10.1164/ajrccm.159.1.ats898 9872857

[B39] Spielberger C. D. (2010). *State-Trait Anxiety Inventory.* New Jersey: John Wiley & Sons, Inc, 10.1002/9780470479216.corpsy0943

[B40] Thomas C. L. Cassady J. C. (2021). Validation of the State Version of the State-Trait Anxiety Inventory in a University Sample. *SAGE Open* 11:21582440211031900. 10.1177/21582440211031900

[B41] Turner D. L. Bach K. B. Martin P. A. Olsen E. B. Brownfield M. Foley K. T. (1997). Modulation of ventilatory control during exercise. *Respir. Physiol.* 110 277–285. 10.1016/s0034-5687(97)00093-5 9407621

[B42] Turner D. L. Sumners D. P. (2002). Associative conditioning of the exercise ventilatory response in humans. *Respir. Physiol. Neurobiol.* 132 159–168. 10.1016/s1569-9048(02)00075-7 12161329

[B43] Turner D L. Stewart J. D. (2004). Associative conditioning with leg cycling and inspiratory resistance enhances the early exercise ventilatory response in humans. *Euro. J. Appl. Physiol.* 93 333–339. 10.1007/s00421-004-1194-2 15375661

[B44] Vitasari P. Wahab M. N. A. Herawan T. Othman A. Sinnadurai S. K. (2011). Re-test of State Trait Anxiety Inventory (STAI) among Engineering Students in Malaysia: reliability and Validity tests. *Proc. Soc. Behav. Sci.* 15 3843–3848. 10.1016/j.sbspro.2011.04.383

[B45] Wasserman K. Whipp B. J. Koyal S. N. Beaver W. L. (1973). Anaerobic Threshold and Respiratory Gas-Exchange During Exercise. *J. Appl. Physiol.* 35 236–243. 10.1152/jappl.1973.35.2.236 4723033

[B46] Weissman M. M. Sholomskas D. Pottenger M. Prusoff B. A. Locke B. Z. (1977). Assessing depressive symptoms in five psychiatric populations: a validation study. *Am. J. Epidemiol.* 106 203–214. 10.1093/oxfordjournals.aje.a112455 900119

